# Sleep Paralysis among Professional Firefighters and a Possible Association with PTSD—Online Survey-Based Study

**DOI:** 10.3390/ijerph18189442

**Published:** 2021-09-07

**Authors:** Paulina Wróbel-Knybel, Joanna Rog, Baland Jalal, Paweł Szewczyk, Hanna Karakuła-Juchnowicz

**Affiliations:** 1 I Department of Psychiatry, Psychotherapy and Early Intervention, Medical University of Lublin, 20-442 Lublin, Poland; joannarog@umlub.pl (J.R.); pawel.szewczyk33@gmail.com (P.S.); hanna.karakula-juchnowicz@umlub.pl (H.K.-J.); 2 Behavioural and Clinical Neuroscience Institute and Department of Psychiatry, University of Cambridge, Cambridge CB2 0QQ, UK; bj272@cam.ac.uk; 3 Department of Clinical Neuropsychiatry, Medical University of Lublin, 20-442 Lublin, Poland

**Keywords:** sleep disorders, sleep paralysis, parasomnias, anxiety, PTSD, firefighters, stress, anxiety disorder

## Abstract

The prevalence of sleep paralysis (SP) is estimated at approximately 7.6% of the world’s general population. One of the strongest factors in the onset of SP is PTSD, which is often found among professional firefighters. Our study aimed to assess in the professional firefighter population (*n* = 831) (1) the prevalence of SP, (2) the relationship between SP and PTSD and (3) the relationship between SP and other factors: the severity of the stress felt, individual tendency to feel anxious and worried and lifestyle variables. The incidence of SP in the study group was 8.7%. The high probability of PTSD was found in 15.04% of subjects and its presence was associated with 1.86 times the odds of developing SP [OR = 1.86 (95% CI: 1.04–3.33); *p* = 0.04]. Officers who experienced at least 1 SP during their lifetime had significantly higher results in the scales: PCL-5, STAI-T, PSWQ. The number of SP episodes was positively correlated with the severity of symptoms measured by the PCL-5, PSS-10, STAI and PSWQ questionnaires. Further research is needed to assess the importance of SP among the firefighter population in the context of mental and somatic health and to specify methods of preventing SP episodes.

## 1. Introduction

Chronic sleep paralysis (SP) is a common but unpleasant experience belonging to parasomnia and can occur when falling asleep or waking up [1]. During its duration, motor function is inhibited, while consciousness remains active [2,3,4]. Some muscles, e.g., respiratory and oculomotor muscles, are not affected, so that a person experiencing SP can breathe and move their eyes [5]. During SP visual, auditory, sensory and kinesthetic hallucinations often occur [6,7]. Usually, the experience of SP is accompanied by strong psychosomatic sensations such as palpitations, chest tightness and increased anxiety or even fear of dying from the event [7].

It is believed that this disorder is related to the abnormal overlap between the REM sleep phase (rapid eye movement sleep) and the waking state [8]. The term Isolated Sleep Paralysis (ISP) is used when episodes of SP are not a symptom of any other medical conditions (e.g., narcolepsy, seizure disorders, substance use) but is an isolated disorder [3]. It is experienced by 7.6% of the general population, although its prevalence varies according to cultural, social and health status [9]. Lifetime prevalence of SP is significantly more common among students, at 28.3%. Among psychiatric patients, the average incidence of ISP is 31.9%, and, for example, in the group of African Americans living in the United States, it is as high as 40.2%.

A high incidence of SP was also observed in people diagnosed with Post-Traumatic Stress Disorder (PTSD), with 27.8–67% [10,11]. In addition, people with other anxiety disorders experience SP more often. SP also develops in 15.8% of people with generalized anxiety disorders and in 22.2% of people with social phobia while in people with panic disorder, depending on the study, it ranged from 20.8% to 30.6% [12]. The relationship between the occurrence of SP and the occurrence of anxiety symptoms has been the subject of many studies, but has not been fully elucidated.

Research shows that as many as 70% of people suffering from PTSD also struggle with sleep disorders. The most common sleep problems in this group of patients include aggressive sleep behavior, sleep talking, hypnagogic and hypnopompic hallucinations and SP [13].

The profession of a firefighter carries a particular risk of PTSD, depression and sleep disorders, due to the considerable physical and mental stress associated with the specificity of this work. It is scientifically proven that health problems such as PTSD, musculoskeletal disorders and heart disease are more prevalent in the firefighter population [14,15]. Research shows that sleep disorders among firefighters are associated with high psychological demands, constant exposure to critical and traumatic situations and stressors of the external environment [16], as well as mental and physical stress or shift work [17].

Depending on the study, sleep disorders among firefighters are estimated at 37–70% [18,19], while in the general population, the frequency of sleep disorders ranges from 15–37% [20,21,22]. The prevalence of PTSD in this profession is also high, amounting to 9.5–31.8% [23,24,25,26], and its presence is associated with a high suicide risk [25].

Despite numerous studies on sleep disorders in the population of fire brigades, the incidence of SP has not been studied so far. The aim of our study was to assess (1) the prevalence of SP, (2) the relationship between SP and PTSD and (3) the relationship between SP and other factors: the intensity of perceived stress, the individual tendency to feel anxiety and worry, and lifestyle variables in the population of professional firefighters.

## 2. Materials and Methods

### 2.1. Study Participants and Procedure

The study used a battery of questionnaires containing open and closed questions. After obtaining the consent of the Commander of the Main Headquarters of the Fire Service of Poland, a request to make the research questionnaire available to firefighter brigades was sent via electronic mail (e-mail) to all Provincial Headquarters of the National Fire Service and all State Schools of Fire Service located in all over Poland. Online research surveys were sent via e-mail by all Provincial Headquarters of the National Fire Services and State Schools of Fire Service in Poland to the professional firefighters working there. Participation in the survey was completely voluntary and anonymous. The inclusion criteria for the study were as follows: (1) written informed consent to participate in this study (acceptance by clicking the button) and (2) working as a firefighter. All data were collected from August 2019 to the end of October 2019.The questionnaire was completed by 831 officers: 27 women (3.35%) and 804 men (96.75%) aged 18 to 51. All participants completed a set of online questionnaires: (1) an authors-made personal questionnaire, (2) the Sleep Paralysis Experience and Phenomenology Questionnaire (SP-EPQ) [27], (3) the PTSD Checklist (PCL-5) [28], (4) the Perceived Stress Scale (PSS-10) [29], (5) the State-Trait Anxiety Inventory (STAI-T) [30] and (6) the Penn State Worry Questionnaire (PSWQ) [31].

The project was approved by the Medical University of Lublin Ethics Committee (the project identification code: KE-0254/125/2017) and performed according to the Declaration of Helsinki guidelines (available online: http://www.nil.org.pl accessed on 18 June 2019).

### 2.2. Materials

#### 2.2.1. Personal Questionnaire

For the current study, a personal questionnaire was developed to collect relevant personal data, lifestyle information and the health status of the study participants. The questionnaire consisted of three parts, containing:

I. Personal data, i.e., gender, age, height, weight.
II. Lifestyle data, i.e., smoking (number of cigarettes smoked during the day, lifetime use of cigarettes measured in pack-year), use of energizing substances (number of cups of coffee during the day), alcohol consumption (frequency of alcohol consumption during the month), the average number of hours of sleep during the day, physical activity (number of hours per week devoted to physical activity).
III. Health data, i.e., the presence of chronic diseases and medications taken.

#### 2.2.2. The Sleep Paralysis Experience and Phenomenology Questionnaire (SP-EPQ)

The SP-EPQ is an updated and expanded version of the Sleep Paralysis Questionnaire (SPQ) by Baland Jalal and Devon Hinton. It assesses the frequency of SP episodes, the presence of psychological and somatic symptoms, prevalence rates and the level of knowledge about this experience [32]. The questionnaire was used in SP studies in Italy, Turkey and Poland [7,27,33,34].

The SP-EPQ consists of 17 open-ended and closed-ended questions regarding the frequency of SP episodes (e.g., life time, last year and month), the average duration of SP episodes, and the emotions experienced during the episodes. The questionnaire also includes elements assessing the nature of the hallucinations during SP, explanations for the cause of SP, and measures taken to prevent subsequent episodes [27]. The first question of the questionnaire is formulated as follows: “Some people have experienced an incident where they couldn’t move their arms, legs, or speak while sleeping or waking up even though they wanted to do so. Have you ever experienced this for yourself?” If the participants answered yes to this question, they were asked to describe the episode, confirming that the experience was in fact SP. Point 8 of the questionnaire consists of 12 closed questions regarding the presence of somatic symptoms during an episode of SP. The calculated value of the Cronbach’s coefficient for this part of the test in our study is 0.83. Cronbach’s alpha indicates that the tests or scales are fit for the performed assessment. Higher scores indicate more reliability of the used tool. The level > 0.9 indicate strong alpha values, level 0.84–0.90 is reliable and the level above 0.70 defines the acceptable threshold [35].

#### 2.2.3. PTSD Checklist (PCL-5)

We used the PTSD Checklist (PCL-5) by Blevins et al. to evaluate the severity of PTSD symptoms [28] in the Polish adaptation of Ogińska Bulik et al. [36]. The tool is designed to study the severity of PTSD symptoms in adults in accordance with the criteria contained in DSM-V. PCL-5 consists of 20 items, relating to 4 subscales, which are re-experiencing, avoidance, negative alterations in cognition and mood and increased arousal and reactivity. The respondent marks answers on a 5-point scale from 0 (not at all) to 4 (very strong). The Cronbach’s alpha coefficient calculated for our study is 0.96.

#### 2.2.4. The Perceived Stress Scale (PSS-10)

The PSS-10 scale we used is a Polish adaptation of the PSS-10 questionnaire [29] and part of the series “Stress Measurement and Stress Management Tools” by Zygfryd Juczyński and Nina Ogińska-Bulik [37]. It is used to measure the perceived stress related to one’s life situation over the past month. It contains 10 questions about different subjective feelings related to problems, stress-related events and ways of coping. The tool is characterized by high accuracy and reliability. The Cronbach’s alpha coefficient calculated for our study is 0.77.

#### 2.2.5. The State-Trait Anxiety Inventory (STAI-T)

In our study, we used the STAI questionnaire by CD Spielberger et al. [38] in the Polish adaptation by K. Wrześniewski et al., 2011 [39]. The tool is designed to study anxiety as a transient and situational state of an individual and anxiety as a relatively constant personality trait. We used the X-2 subscale, which rates anxiety as a personality trait. Research shows that the number of points on the subscale also strongly correlates with the severity of depressive symptoms [40]. The subscale consists of 20 questions. On the Likert 5-point scale, respondents indicate to what extent the behavior described in the question is typical for them. The final score is obtained by adding up all items. The Cronbach’s alpha coefficient calculated for our study is 0.77.

#### 2.2.6. The Penn State Worry Questionnaire (PSWQ)

The PSWQ questionnaire by TJ Meyer et al., 1990, is used to assess the severity of worrying [41]. In our study, we used the Polish version of the PSWQ questionnaire by K. Janowski, 2007 [42]. The questionnaire consists of 16 test items. The respondents give answers on a 5-point scale, indicating how typical the behavior described by a given statement is for them. The answers range from 1 (not typical for me at all) to 5 (very typical for me). The theoretical minimum rating is 16 and the maximum is 80, with higher scores indicating a greater tendency to worry. The method has very good psychometric properties and is the most frequently used tool to measure the severity of worrying in the world. The Cronbach’s alpha coefficient calculated for our study is 0.77.

#### 2.2.7. Statistical Analysis/Data Analysis

Prior to the obtained data analysis, the internal consistency of the used scales was assessed by determining Cronbach’s alpha.

A descriptive analysis was performed to characterize the study sample expressed by frequencies and measured of central tendency (i.e., median (M), mean (X) and standard deviation (SD)). We applied a Shapiro–Wilk test to assess the distribution of quantitative data. A chi-squared test (χ2) to compare categorical variables was applied. Differences between examined factors were analyzed through the Mann–Whitney U test and the relationship between not normally distributed continuous and ordinal variables was determined using Spearman’s rank-order correlation test.

We performed univariate logistic regression and calculated the odds ratio with 95% confidence interval (CI) to find significant factors associated with sleep paralysis presence. Stepwise logistic regression analysis was conducted to evaluate factors associated with sleep paralysis. The significance of logistic coefficients was tested using the Wald test. For analyses, *p* ≤ 0.05 was considered statistically significant.

The statistical analysis was carried out with using Statistica (STATISTICA, version 12; StatSoft, Inc., Tulsa, OK, USA).

## 3. Results

### 3.1. Demographic Characteristics and Health Status of Respondents

The demographic characteristics and health status of the respondents are presented in Table 1.

### 3.2. SP Incidence

Of the 831 National Fire Service officers participating in the study, 72 respondents (8.7%) experienced at least one SP episode in their lifetime (14.8% F, 8.5% M). Thirteen participants experienced at least one episode in the last month, while 11 experienced 4 or more SP episodes in the past year. For officers who experienced at least one SP episode, the mean lifetime number of SP episodes was 9.57.

### 3.3. The Relationship between SP and PTSD

#### 3.3.1. SP and Severity of Symptoms of Post-Traumatic Stress Disorder (PTSD)

It was found that firefighters who had at least one episode of SP during their lifetime had a higher intensity of PTSD symptoms (they obtained a higher score on the PCL-5 questionnaire) compared to those who never experienced SP: M = 21.35, Me = 19.0, SD = 19.39 vs. M = 15.40, Me = 12.0, SD = 14.62 (Z = 2.29; *p* = 0.02). The results are shown in Table 2.

It was also shown that officers who experienced at least one SP episode had significantly higher scores on two of the four subscales of the PCL-5 questionnaire (1—signs of intrusion; 2—signs of increased agitation and reactivity) compared to those who never experienced SP. The results are shown in Table 2.

A positive correlation was found among the respondents between the number of SP episodes in the past month (R = 0.35; *p* < 0.05), past year (R = 0.52; *p* < 0.05) and one’s lifetime (R = 0.33; *p* < 0.05) and the severity of PTSD symptoms (number of points on the PCL-5 questionnaire). A positive correlation between the number of SP episodes in the past month, year and whole life and the severity of PTSD symptoms measured on each of the 4 subscales of the PCL-5 questionnaire. The results are shown in Table 3.

#### 3.3.2. Prevalence of SP in Officers with a High Probability of PTSD (PCL-5 ≥ 33 points)

Taking the cut-off point established for PCL-5 (33 points), it can be indicated that 125 people, i.e., 15.04% of respondents, show a high probability of PTSD.

In the studied group of participants, a significantly higher frequency of SP during the past month, year and whole life was observed in officers with a high probability of having PTSD compared to those with a low probability of having PTSD. The results are shown in Table 4.

#### 3.3.3. Chance of Occurrence of SP in Officers with a High Probability of PTSD (PCL-5 ≥ 33 Points)

In the group of officers who experienced at least one SP episode, a high probability of PTSD concerns 23.61% (*n* = 17), while in the group of officers who had never experienced SP, a high probability was found at 14.23% (*n* = 108)

Study participants who had a high probability of having PTSD (PCL-5 ≥ 33 points) have 1.86 times the odds of developing SP [OR = 1.86 (95% CI: 1.04–3.33); *p* = 0.04] compared to people with no significant probability of PTSD.

### 3.4. SP and the Intensity of Perceived Stress (PSS-10)

A positive correlation was found among the respondents concerning the number of SP episodes in the past month (R = 0.31; *p* < 0.05), year (R = 0.33; *p* < 0.05) and lifetime (R = 0.26; *p* < 0.05) and the increase in perceived stress (number of points on the PSS-10 questionnaire). The results are shown in Table S1.

### 3.5. The Relationship between SP and the Individual Tendency to Feel Anxious and Worried

#### 3.5.1. SP and Individual Tendency to Feel Anxiety

Among the surveyed firefighters, a higher level of anxiety as a constant personality trait (number of points in the STAI questionnaire ≥ 41) was associated with 2.19 times the odds of SP [OR = 2.19 (95% CI: 1.34–3.56); *p* = 0.001]

Participants who experienced at least one SP episode during their lifetime had significantly higher levels of personality trait anxiety compared to those who had never experienced it: M = 41.17, Me = 42.0, SD = 11.94 vs. M = 37.29, Me = 36.0, SD = 8.96 (Z = 2.70; *p* = 0.006).

There was a positive correlation between the number of SP episodes in the past month (R = 0.26; *p* < 0.05), year (R = 0.33; *p* < 0.05) and lifetime (R = 0.31; *p* < 0.05) and the severity of anxiety as a constant personality trait (number of points in the STAI questionnaire). The results are shown in Table S1.

#### 3.5.2. SP and a Tendency to Worry

Higher tendency to worry (number of points in the PSWQ questionnaire ≥ 46) was associated with 1.85 times the odds of developing SP [OR = 1.85, (95% CI: 1.14–3.0); *p* = 0.013] in the group of tested firefighters.

Participants who had at least one SP episode during their lifetime had a higher tendency to worry (they scored higher on the PSWQ) compared to those who never experienced SP: M = 46.13, Me = 46.0, SD = 13.00 vs. M = 42.20, Me = 42.0, SD = 10.33 (Z = 2.17; *p* = 0.03).

Among the respondents, a positive correlation was found between the number of SP episodes in the past month (R = 0.24; *p* < 0.05) and year (R = 0.23; *p* < 0.05) and the tendency to worry (number of points in the PSWQ questionnaire). The results are shown in Table S1.

### 3.6. Lifestyle and SP Occurrence

Significant lifestyle differences between firefighters who experienced at least one SP episode and those who never experienced it are shown in Table 5.

### 3.7. Predictors of Sleep Paralysis in Multiple Stepwise Regression Model

Table 6 depicts the results of stepwise regression analysis. After adjustment for confounders (all factors indicate in Table 5.), BMI [OR = 0.80 (95% CI: 0.73–0.89)], average sleep time during the night [OR = 0.69 (95% CI: 0.56–0.86)] and the frequency of alcohol consumption [OR = 1.30 (95% CI: 1.08–1.58)] remained significant predictors of sleep paralysis.

## 4. Discussion

The scope of our study concerned the population of National Fire Service officers, who are particularly vulnerable to sleep disorders as well as psychological problems, such as PTSD, occupational burnout, chronic stress and depression [16,43].

The prevalence of sleep paralysis (which can constitute a sleep disorder when it becomes chronic) turned out to be higher in the firefighter population than the global average (8.7% vs. 7.6%) [9]. The prevalence of SP in the firefighter population is relatively high, considering the fact that the prevalence of this disorder in general populations of countries similar to ours in social and cultural terms is significantly lower, as exemplified by Germany (2.3%), Spain, Italy (6.2%), and Canada (2.4%) [9,13,44,45].

Our results indicate that an initial high probability of PTSD is associated with 1.86 times the odds of developing SP. Moreover, those with a PCL-5 questionnaire result indicating PTSD had a higher frequency of SP episodes compared to those whose results in PCL-5 were below the cut-off threshold allowing for the diagnosis of PTSD. Firefighters who experienced at least one episode of SP in their lifetime also had greater intensity of PTSD symptoms and a higher individual tendency to feel anxious and worried compared to those who had never experienced SP. Other researchers have also noted that SP may be associated with trauma and anxiety. Research indicates that the number of traumatic experiences is positively correlated with the number of SP episodes in life [46,47]. Hinton et al., 2005, noted that the prevalence of SP among Cambodian refugees diagnosed with PTSD was as high as 67% [11]. Numerous studies have confirmed a higher prevalence of SP among patients with a clinical diagnosis of anxiety disorders, such as Panic Disorder and Generalized Anxiety Disorder, or affective disorders, such as Affective Seasonal Disorder [12,48,49]. Baland Jalal and Devon Hinton showed that students who experienced at least one SP in their lifetime were characterized by a higher level of anxiety as a trait, a greater tendency to pathologically worry and a higher severity of PTSD symptoms [27]. So far, no studies have been conducted on the incidence and predictors of SP in firefighter populations or other occupations exposed to above-average stress. Based on the results of our study, it can be hypothesized that the occurrence of SP is more common in firefighters with higher levels of anxiety as a persistent personality trait and that PTSD may be a contributing factor to SP and one of its symptoms. Research indicates that complaints about symptoms of REM sleep phase disturbance may indicate an underlying problem of PTSD [13,50]. However, the causal relationship between SP and PTSD remains unclear, especially given our stepwise regression results. It is also worth noting that the individual tendency to feel anxiety, measured with the STAI-T questionnaire, according to the current scientific knowledge, strongly correlates with both the intensity of anxiety as well as symptoms of depression. It is proposed to treat STAI-T as a nonspecific measure of negative affectivity, not trait anxiety [40]. Therefore, it can be concluded that SP episodes increased negative affect and worry/anxiety may be a manifestation of PTSD.

De Barros et al., 2013, showed that the high rate of sleep disorders among firefighters may be related to the physical and psychological stress to which they are exposed while on duty [51]. Our results confirm De Barros’ observations, as the number of SP episodes was positively correlated with the intensity of stress during the last month on the PSS-10 scale. In the studies conducted so far, a similar relationship has also been noticed, because people who experienced SP had significantly higher levels of stress, as measured by self-report scales, compared to those who had never experienced SP [46,52,53].

Despite the lack of literature dealing with the problem of SP in the profession population exposed to chronic stress, several studies have been conducted on sleep disorders in general among firefighters and police officers. According to research, many firefighters complain of poor sleep quality or sleep disturbances. In the study by Abbasi et al., 2018, as many as 59% of firefighters complained about poor sleep quality [43], while other sources show that, depending on the study, 37–70% of firefighters suffer from sleep disorders [17,18,51,54]. Importantly, Barger et al., 2012, note that more than 80% of surveyed firefighters who met the criteria for sleep disorders had never been diagnosed and treated [17]. In comparison, among police officers, 23% to 79% experience poor sleep quality [55,56], and about 40.4% [56] have been diagnosed with sleep disorders, which is significantly less than among firefighters. Moreover, as research shows, sleep disorders in firefighters are a risk factor for hypertension and strokes [17]. Given this, it would be extremely important to investigate the impact of SP on the somatic health of firefighters, especially considering the likely contribution of sleep to the risk of developing sleep paralysis. Interestingly, longer sleep time was a factor associated with the emergence of sleep paralysis, even after adjusting the model to psychological and lifestyle variables. Apart from sleep duration, other lifestyle factors that significantly increased the risk of SP in our study group were lower BMI and a higher frequency of alcohol consumption per month. The current literature on SP does not confirm the relationship of SP with BMI, except for the study of Sharpless et al., 2010, which found a positive correlation between the amount of FISP (Fearful Isolated Sleep Paralysis) and the BMI of the subjects [57]. Similarly, there are little data on the association of SP with alcohol consumption. Munezawa et al. found a higher frequency in adolescents who drink alcohol compared to those who never drink alcohol [58]. Since many of the studies conducted so far have failed to account for lifestyle factors as predictors of the emergence or maintenance of SP, further research is needed. It also seems important to take into account the interactions between psychological variables and behaviors affecting health.

The strengths of the study are its large cohort group and the extensive number of variables included in the personal questionnaire. The online form of the survey precluded “interviewer error” and also provided the respondents with comfort and sufficient time whilst completing the survey.

The inherent limitations of the online study prevent the participants from having direct contact with the researcher who otherwise would be able to address an issue concerning the survey or its process; therefore, conditions for conducting the survey could not be controlled (lack of control over external factors that could distract respondents and skew results). Due to this, recall and desirability biases may occur. Furthermore, the tests were self-reported, which could have influenced the results of the study. It should be noted that a limited number of research methodologies were employed in the study of anxiety and PTSD symptoms and in the future, it would be worthwhile to expand them. It also seems worthwhile to expand the study to include affective symptoms, ways of coping with stress and employ a more thorough assessment of quality of sleep and severity of sleep disorders. In our study, we did not ask participants about any history of concussion or head traumas, which could be relevant to sleep disorders such as SP. Another methodological limitation of our study is that the “Sleep Paralysis Experience and Phenomenology Questionnaire” was not validated in Polish, and was recently used in a previous work without a validation procedure. It should be mentioned that the cross-sectional nature of the study prevents determining the causality. Further studies should be performed to confirm the results.

Our discovery seems to be particularly important in that problems with sleep and mental health are associated not only with an increased risk of burnout in firefighters [59], but also with the development of somatic diseases [17]. We speculate that officers’ experiences of SP may be directly related to the presence of other health problems such as PTSD, anxiety or depressive disorders. Moreover, the occurrence of SP episodes may be one of the symptoms of these disorders, often one of the first to be noticed by the patient and an indication to start searching for a more comprehensive diagnosis. Knowledge about SP is lacking not only among people working in professions particularly exposed to psychological trauma and higher than average stress levels, but also in the general population of Poland. It would be important to develop special guidelines for patients wherein they could report SP events for the purpose of diagnosing an underlying occult anxiety or depression disorder and ultimately receive proper psychological and therapeutic help. Especially important would be to increase the public’s awareness of sleep and anxiety disorders by providing better education and access to special psychological care for professional and social groups at risk of depressive disorders or post-traumatic stress disorder due to occupation-related chronic stress and anxiety.

## 5. Conclusions

The prevalence of SP in the National Fire Service surveyed was higher than in the general population (8.7% vs. 7.6%). Our research statistically reveals a link between SP and PTSD. Officers suffering from PTSD had 1.86 times the odds of developing SP compared to healthy controls. People with PTSD had a higher frequency of SP episodes compared to healthy people.

The incidence of SP was positively correlated with both the severity of PTSD symptoms and the symptoms of an individual tendency to feel anxiety and worry, and with a greater intensity of perceived stress. The high number of points in the STAI and PSWQ questionnaires significantly increased SP risk. Moreover, significant predictors of SP were BMI, average sleep duration and frequency of alcohol consumption. Further research is needed to assess the importance of SP among the firefighter population in the context of mental and somatic health and to specify methods of preventing SP episodes.

## Figures and Tables

**Table 1:** Participants’ demographic and health status characteristics.

Sample Type		SP +	SP −
*N*		72	759
Female *N*		4	23
Male *N*		68	736
Age	*M* (*SD*)	34.5 (6.82)	36 (7.64)
	RNG	23–51	18–50
BMI (kg/m^2^) Z = 4.06 ***	M (SD)	25.14 (2.58)	26.66 (3.15)
	Me	24.97	26.29
	RNG	19.38–33.51	17.93–50.04
Psychiatric Disorder *N*	All	1	5
	Depression	1	2
	PTSD	-	1
	Alcohol addiction	-	1
	Insomnia	-	1
Somatic disorder *n*	All	7	58
	Endocrine Disorders	1	7
	Hypertension	3	25
	Allergic and Atopic Disorders	1	6
	Gastrointestinal Disorders	1	5
	Musculoskeletal disease	1	7
	Others (*n*)	-	Sarcoidosis (1), Myopia (1), Deafness (1), Lyme disease (1), Glaucoma (1), chronic sinusitis (1), Psoriasis (1), Anemia (1)
Medicines taken (medications taken on a permanent basis) *n*	All	7	54
	Psychiatric/hypnotic medications (*n*)	Sertraline (1)	sertraline + lithium (1), opipramol and escitalopram (1), mianserin (1), trazodone + citalopram (1)

Note SP+ = participants with at least one lifetime episode of SP; SP− = individuals who have not experienced SP; SD = standard deviation; M = mean; Me = Median; RNG = refers to range; Z = score; *p* = significance coefficients *** *p* < 0.001.

**Table 2:** Comparison of self-report measures for firefighters who experienced at least one SP in their lives and those who never experienced SP.

	SP+			SP−			
Self-Report Measures	**Me**	***M***	**(SD)**	**Me**	***M***	**(SD)**	***Z***
PCL overall score	19.0	21.35	(19.39)	12.0	15.40	(14.62)	2.29 *
PTSD—Re-experiencing	3.0	5.01	(5.19)	2.0	3.43	(3.91)	2.39 *
PTSD—Increased arousal and reactivity	7.0	7.54	(6.19)	5.9	5.57	(4.98)	2.48 *

Note. SP+ = participants with at least one life time episode of SP; SP− = individuals who have not experienced SP; Me = median; M = mean; SD = standard deviation; Z = score; *p* = significance coefficients * *p* < 0.05.

**Table 3:** Correlation between PTSD symptoms and the number of SP episodes.

Number of Points in the PCL-5 Scale and Four Subscales	PTSD—Overall Score	PTSD—Re-experiencing	PTSD—Avoidance	PTSD—Negative Alterations in Cognition and Mood	PTSD—Increased Arousal and Reactivity
Number of SP episodes in the past month	0.35	0.34	0.28	0.37	0.28
Number of SP episodes in the past year	0.52	0.53	0.44	0.46	0.49
Number of SP episodes in one’s lifetime	0.33	0.26	0.27	0.31	0.37

Note. The correlation between the number of SP episodes and the number of points in the PCL-5 scale and four subscales corresponding to the four symptom clusters is presented using Spearman’s rank correlation coefficient (rs). Significance coefficients, *p* < 0.05.

**Table 4:** Comparison of frequency of SP episodes between firefighters who meet PTSD criteria and those who do not meet PTSD criteria.

	PTSD+ (*n* = 17)			PTSD− (*n* = 55)			
Number of SP Episodes:	Me	*M*	(SD)	Me	*M*	(SD)	*Z*
In the past month	0	0.59	(0.87)	0	0.18	(0.58)	2.71 **
In the past year	3.0	4.24	(4.84)	0	1.29	(3.06)	3.67 ***
In one’s lifetime	10.0	15.18	(22.99)	4.0	7.84	(10.22)	1.98 *

Note. PTSD+ = firefighters with at least one lifetime episode of SP and meet PTSD criteria (PCL-5 ≥ 33 points); PTSD− = firefighters with at least one lifetime episode of SP and does not meet PTSD criteria (PCL-5 < 33 points); Me = median; M = mean; SD = standard deviation; Z = score; *p* (significance coefficients) *** *p* < 0.001, ** *p* < 0.01, * *p* < 0.05.

**Table 5:** Lifestyle differences between firefighters who experienced at least one SP in their lives and those who never experienced SP.

	Professional Firefighters						
	SP+			SP−			
	Me	M	(SD)	Me	M	(SD)	Z
BMI (kg/m^2^)	24.97	25.14	(2.58)	26.29	26.66	(3.15)	4.06 ***
Cigarette smoking (number of cigarettes smoked during the day)	0	1.81	(4.99)	0	2.26	(6.20)	−0.16
Lifetime use of cigarettes (pack-years)	0	0.16	(0.36)	0	0.15	(0.36)	−0.13
Coffee consumption (number of cubs during the day)	2.0	1.93	(1.40)	2.0	1.61	(1.34)	−2.04 *
Alcohol consumption (frequency within a month)	3	2.58	(1.28)	3.0	2.25	(1.38)	−2.17 *
Average sleep time during the night (hours)	7.0	6.61	(1.08)	7.0	7.14	(1.12)	3.99 ***
Physical activity (number of hours per week devoted to physical activity)	3.0	3.22	(2.49)	3.0	2.83	(2.54)	−1.49

Note. SP+ = participants with at least one lifetime episode of SP; SP− = individuals who have not experienced SP; BMI = Body Mass Index; Me = median; M = mean; SD = standard deviation; Z = score; *p* = significance coefficients *** *p* < 0.001, * *p* < 0.05.

**Table 6:** Predictors of sleep paralysis in multiple stepwise regression model.

	OR	95% CI	Wald Test	*p*-Value
STAI * PSWQ	1.00	1.00–1.01	11.14	0.001
BMI	0.80	0.73–0.89	18.93	<0.001
Average sleep time during the night	0.69	0.56–0.86	11.50	0.001
Frequency of alcohol consumption	1.30	1.08–1.58	7.37	0.007

STAI—The State-Trait Anxiety Inventory; PSWQ—The Penn State Worry Questionnaire; BMI—body mass index; *—interaction between variables; OR—odds ratio; CI—confidence interval.

## Data Availability

The datasets generated and/or analyzed during the current study are not publicly available but are available from the corresponding author on reasonable request.

## Supplementary Materials

The following are available online at https://www.mdpi.com/article/10.3390/ijerph18189442/s1, Table S1: Correlation between anxiety symptoms and the number of SP episodes.
