# Supplementary material for: Sleep Paralysis among Professional Firefighters and a Possible Association with PTSD—Online Survey-Based Study
Source: Int J Environ Res Public Health. 2021 Sep 7;18(18):9442. doi: 10.3390/ijerph18189442 (PMC8468000; doi:10.3390/ijerph18189442)
Supplement: Supplementary file 1 [file ijerph-18-09442-s001.zip › ijerph-1318204-supplementary.pdf]

Table S1. Correlation between anxiety symptoms and the number of SP episodes.

Self-Report Measures	PCL	STAI-T	PSWQ	PSS-10
Number of SP episodes in the last month	0.35	0.26	0.24	0.31
Number of SP episodes in the last year	0.52	0.33	0.23	0.33
Number of SP episodes in one's lifetime	0.33	0.31	-	0.26

Note. The correlation between anxiety symptoms and the number of SP symptoms is presented using the Spearman's rank correlation coefficient (rs). Significance coefficients, $p < 0.05$.
